# Use of haemostatic matrix in management of rectus hematoma in an anticoagulated patient: a case report

**DOI:** 10.4076/1757-1626-2-6353

**Published:** 2009-07-07

**Authors:** Kansav Tunc Kizilkanat, Engin Olcucuoglu, Hakan Kulacoglu

**Affiliations:** Department of Surgery, Diskapi Yildirim Beyazit Teaching and Research HospitalAnkaraTurkey

## Abstract

Rectus sheath hematoma may present as a painful mass in the anterior abdominal wall. The underlying reasons may vary, while anticoagulant use and thromboembolism prophylaxis are documented causes. Treatment is mostly conservative however interventional procedures can be required. We herein present a case of 76-year-old patient with an uncontrolled rectus hematoma despite surgical hemostasis. The patient was treated succesfully by applying hemostatic matrix (Floseal).

## Introduction

Rectus sheath hematoma may present as a painful lesion with or without a visible mass on the anterior abdominal wall. The underlying reasons may vary. Etiology may consist of any kind of trauma including abdominal surgery or even a strenuous cough [1]. Oral anticoagulant usage and thromboembolism prophylaxis with heparins are also well-documented causes of rectus sheath hematoma [2]. The treatment of rectus sheath hematoma is conservative in many cases [2-5] however interventional procedures can be required in some cases [4,5].

## Case presentation

**Figure 1.**
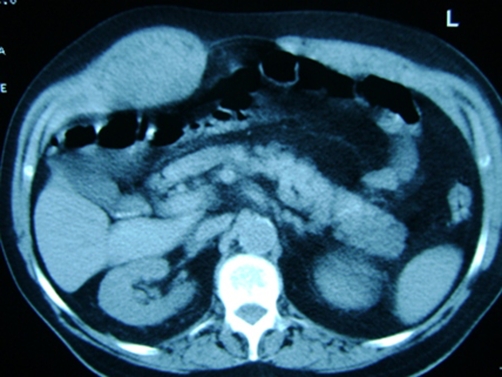
Computed tomography displayed a 10 × 5 cm mass at the right side of the abdominal wall; possible rectus hematoma.

A 76-year-old retired Caucasian Turkish male patient admitted to the emergency department with a rapid onset painful mass in the abdominal wall. The mass was in 15 cm in diameter and very tender on physical examination, and computed tomography displayed a 10 × 5 cm. hematoma (Figure 1). Hemoglobin level was 14.2 gm and hematocrit 42.8%. Patient's history revealed oral anticoagulant use [Acetylsalicylate 300 tablet daily] after a successful coronary by-pass surgery 10 years ago and cardiac pacemaker placement 2 years ago. Surgical exploration via a vertical incision over the mass under general anesthesia in the emergency operating room displayed a large and expanding rectus muscle hematoma. Hemostasis was applied with compression and ligation. The incision was closed after leaving a suction drain in situ.

**Figure 2.**
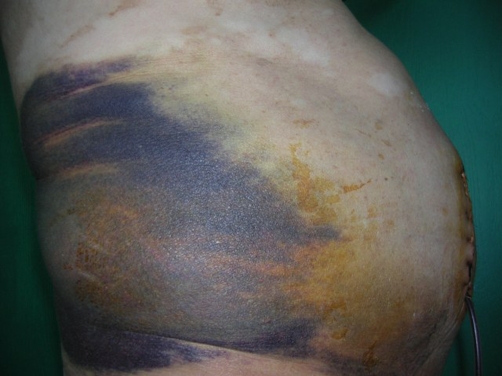
Continuing hemorrhagic drainage and a massive ecchymosis on right side in spite of primary surgical hemostatis.

**Figure 3.**
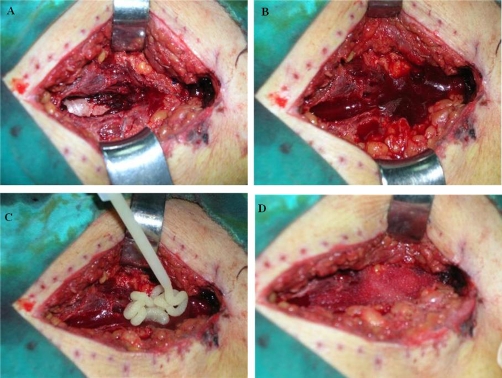
(**A**) Re-exploration and initial aspiration of the field: rectus muscle were torn to display posterior sheet. (**B**) Immediate re-bleeding. (**C**) Application of hemostatic matrix after preparation. (**D**) Granules covered the cavity to stop bleeding.

The patient developed a large right lumbar ecchymosis while daily drainage volume remained 150-200 ml (Figure 2). Hemoglobin level dropped to 8.9 g and hematocrit to 26.6%. He was still complaining of considerable pain in spite of medications. A re-exploration was decided on day-5. Previous incision was re-opened with local anesthetic infiltration. A pulsatile hemorrhage was seen from an approximately 1-mm vessel. Despite this vessel was ligated the hemorrhage kept continuing. The operating team concluded to try hemostatic matrix [FloSeal®, Baxter] for definitive hemostasis. After applying this novel hemostatic agent the bleeding stopped (Figure 3).

Daily drainage was 20 and 10 ml. on day-1 and -2. Ecchymosis started to fade away. Patient complained no pain. Drain was removed after 48 hours and the patient was discharged. Control examinations on 3, 7, and 15 day after were completely alright.

## Discussion

The treatment of rectus sheath hematoma is mostly conservative [1-4] however surgical interventions including laparotomy [5] or endovascular embolizations have been reported in patients develop clinical deterioration [4]. This is especially the case for some patients on anticoagulant use which may cause life-threatening abdominal wall hematomas especially when concomitant systemic disorders also exist [2].

**Figure 4.**
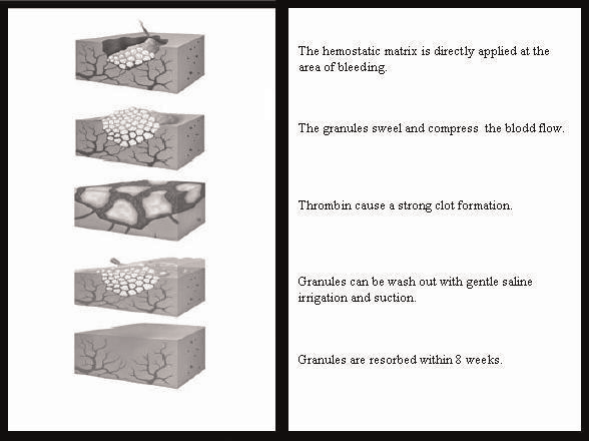
Mechanism of action in direct application.

The novel agent FloSeal consists of a bovine-derived gelatin matrix component and a human-derived thrombin component. It works on wet, actively bleeding tissue and conforms to irregular wound surfaces (Figure 4). It has been proven to control bleeding from oozing to pulsatile flow [6]. Like the other exogenous hemostatic agents Floseal are finding increased applications in surgery. Following its first clinical use reported in 2000 from United States, the spectrum has been enlarged from case reports in different disciplines [7,8] to randomized controlled studies in otolaryngology [9,10].

The use of hemostatic matrix in general surgery field is rather limited with sporadic applications in patients suffered abdominal traumas [11]. Very recently, Izzo et al. reported a large prospective study of 237 consecutive patients undergoing major hepatic surgery and stated that the application of a hemostatic matrix provided rapid and effective intraoperative control of mild to severe bleeding from the liver edge, even in patients with prolonged bleeding times resulting from cirrhosis [12]. To the best of our knowledge no report has been published to date about the use of hemostatic matrix in rectus muscle hematoma or other abdominal wall injuries. Regarding in-muscle tissue experience, there is only one report describing the use of this matrix in successful repair of iatrogenic diaphragmatic injury during laparoscopy instead of a formal suture repair [13]. It has been shown experimentally that hemostatic matrix has no adverse effect on the function of the neurovascular bundles [14]. This may be a clue for its safe use in muscles which need an intact neurovascular supply.

In fact, hemostatic matrix is a quite expensive commercial material. It is more logical to use it in major surgical procedures and acute life-threatening hemorrhages. However its application may also be valuable and in some moderately critical patients with severe concomitant diseases and coagulation disorders to avoid long hospital stays, longer immobilization and higher in-hospital care cost.

## Abbreviations

None
